# The Impact of Comorbidities and Motor Impairment on the Quality of Life of Patients with Spinal Muscular Atrophy: A Case–Control Study

**DOI:** 10.3390/jcm13144184

**Published:** 2024-07-17

**Authors:** Małgorzata Błauciak, Jakub Ubysz, Anna Pokryszko-Dragan, Magdalena Koszewicz

**Affiliations:** 1 Clinical Department of Neurology, University Centre of Neurology and Neurosurgery, Faculty of Medicine, Wroclaw Medical University, Borowska 213, 50-556 Wroclaw, Poland; malgorzata.blauciak@student.umw.edu.pl (M.B.); jakub.ubysz@student.umw.edu.pl (J.U.); anna.pokryszko-dragan@umw.edu.pl (A.P.-D.); 2 Clinical Neurophysiology Laboratory, University Centre of Neurology and Neurosurgery, Faculty of Medicine, Wroclaw Medical University, Borowska 213, 50-556 Wroclaw, Poland

**Keywords:** spinal muscular atrophy, comorbidities, internal medicine, quality of life, WHOQOL-BREF questionnaire

## Abstract

**Introduction**: Spinal muscular atrophy (SMA) is a genetically determined disease primarily leading to muscle weakness, but now, it is considered a systemic disease with changes in various tissues and organs. In our study, we aimed to compare quality of life (QoL) outcomes in patients with SMA in relation to the degree of motor limitation and comorbidities, mainly internal medicine diseases. **Methods**: We included 35 adult patients with SMA and 36 healthy volunteers. Thorough medical histories were taken focusing on comorbidities, and neurological examinations incorporating assessments using functional motor scales were performed. QoL was assessed based on the World Health Organization Quality of Life Brief Version (WHOQOL-BREF) questionnaire. **Results**: SMA patients and controls were comparable in terms of scores in the questionnaire’s main domains. SMA patients presented significantly higher levels of satisfaction with their medical care than controls. Patients with more advanced SMA had significantly better scores on certain questions, e.g., those related to health satisfaction or leisure activities. A total of 71.4% of SMA patients had comorbidities, ranging from one to three in individual patients. SMA patients with comorbidities did not show worse QoL. Negative correlations were found between the number of comorbidities in SMA patients and individual questions on the WHOQOL-BREF questionnaire. **Conclusions**: Patients with SMA were satisfied with their medical care. Better scores on some questions in more advanced SMA may have been due to better adaptation to disease-related limitations. The presence of single comorbidities did not affect QoL, but a higher number of comorbidities negatively correlated with QoL.

## 1. Introduction

Spinal muscular atrophy (SMA) is a genetically determined disease that leads to impaired muscle function and problems with mobility. Often, the degree of disability is severe. Some patients are unable to walk or sit up unaided and are bedridden. The most severe forms of SMA (type 1) involve people who have never achieved the ability to sit up unaided; patients with type 2 SMA have not achieved the ability to walk; in type 3 SMA, patients are able to walk but lose this ability as the disease progresses. The above division of SMA has been a historical one due to the therapeutic options dramatically changing the progression of the disease [1,2,3]. A significant degree of disability is always an aggravating factor and significantly impairs the quality of life (QoL) of patients. The results of published studies to date involving SMA patients and their carers show that QoL is related to the degree of disability [4,5]. Before treatment, QoL strongly correlates with motor function scores on the Hammersmith Functional Motor Scale Expanded (HFMSE) and Revised Upper Limb Module (RULM). These parameters also correlate negatively with high caregiver burden [4,6,7]. QoL does not increase significantly during the treatment period despite the improvement in motor function [6,8]. Zumani et al. [5] did not observe a significantly lower QoL in a group of children with type 2 SMA in relation to patients with type 3, although it was lower in relation to healthy people. In a German pediatric population with SMA, Landfeldt et al. [4] showed that despite significant physical disability, children had surprisingly good self-assessed health-related QoL as assessed by the KIDSCREEN-27 scale. Based on the studies cited above, it is still not possible to clearly determine the degree of reduction in QoL in the population of SMA patients. QoL appears to be disproportionately high in relation to the degree of patients’ disability and life limitations, including occupational limitations and basic activities of daily living.

Numerous studies on SMA undertaken primarily in connection with available or future therapies have provided the basis for the thesis that SMA is a multi-system disease [9,10] and that the SMN protein plays multiple roles in cell metabolism [11,12]. It is important not only for alpha motoneuron function but also for other cells in the human body [12,13]. Clinical observations of groups of SMA patients have shown the presence of various disorders resulting in abnormal metabolism, including RNA metabolism, glucose, lipids, the calcium–phosphate system, etc. [11,14,15]. Disorders relative to the central nervous system [16], as well as the cardiovascular [17], gastrointestinal [18,19], and genitourinary [20] systems, have been described in SMA patients. Addressing the issue of assessing the QoL of SMA patients in the context of comorbidities appears to be a modern approach that considers the multi-organ significance of SMN protein deficiency.

Given that multimorbidity can have a significant negative impact on patients’ clinical status and QoL, we aimed to compare QoL outcomes in a group of SMA patients in relation to factors discriminating their neurological clinical condition, especially the degree of motor limitation, and in relation to comorbidities, mainly internal medicine disorders.

## 2. Materials and Methods

We obtained the approval of the Ethics Committee of Wroclaw Medical University in Poland. All participants gave their written informed consent. This study was conducted in accordance with the principles of good clinical practice (GCP).

We included 35 adult patients (20 females and 15 males) with SMA and 36 healthy volunteers (18 females and 18 males). Mean age was similar in both groups (35.3 ± 11.6 in the SMA group and 35.1 ± 10.53 in the controls). SMA was confirmed by genetic testing with the estimation of the number of gene copies. We assessed the patients with SMA type 2 or 3 before entering a treatment program with nusinersen or risdiplam.

General clinical and neurological examinations were performed in all patients. They were assessed by using commonly used scales [21]: the Hammersmith Functional Motor Scale Expanded (HFMSE) and the Children’s Hospital of Philadelphia Infant Test of Neuromuscular Disorders (CHOP-INTEND). The CHOP-INTEND scale was used in 19 patients unable to walk independently. Table 1 shows basic data (demographics, number of gene copies, and scale scores) in the group of patients with SMA types 2 and 3. In basic laboratory testing, e.g., morphology, basic biochemistry and creatinine and creatine kinase (CK) levels were assessed.

In all patients, a thorough medical history was taken regarding comorbidities. Patients’ own medical records were used. Based on this and on the results of tests conducted during hospitalization to qualify patients for the therapeutic program, the diagnoses of comorbidities were established.

We assessed QoL in the group of patients with SMA and in the control group based on the World Health Organization Quality of Life Brief Version (WHOQOL-BREF) questionnaire (Polish language version) [22,23]. The English language version is included in Table 2.

The WHOQOL-BREF questionnaire is commonly used to assess the quality of life of both healthy and sick people in clinical practice. It describes quality of life in four domains: physical, mental, social, and environmental functioning [22]. The results of the WHO-QOL-BREF questionnaire in the study and control groups were compared in terms of 26 specific questions and in terms of physical, psychological, social, and environmental domains. In the next step, we compared the questionnaire results in patients with SMA and the comorbidities listed above with patients diagnosed with SMA without any comorbidities and the controls.

The results were analyzed statistically. The continuous data are presented as mean values and standard deviation (SD) when they follow a normal distribution, and median (M) and interquartile ranges (Q1–Q3) were used when they do not follow the normal curve. The categorical data were presented in absolute values and percentages (%). The normality assumption was assessed with the Shapiro–Wilk test. The homogeneity of variances was assessed by using Levene’s test. For the comparisons of variables between the studied groups with normal distribution, the Student’s *t*-test was used. The comparison of variables with non-parametric distribution was conducted with the Mann–Whitney U test. Correlation analysis was performed by calculating the Spearman correlation coefficient (R). A *p*-value ≤ 0.05 was considered significant for all tests. Statistical analysis was performed by using STATISTICA 13.3 software.

We first compared the results of the questionnaire for the SMA patient group, distinguishing SMA type, SMN gene copy number, walking ability, HFSME scores, and CK levels as an indicator of muscle damage, and the control group. Then, we distinguished subgroups with different comorbidities in the SMA group, and a comparison was made between them. We performed an additional statistical analysis in the SMA group on the impact of scoliosis/severe scoliosis on patients’ QoL as an effect of an additional complication probably directly related to the underlying disease [24].

## 3. Results

### 3.1. Clinical Data

The CK levels were normal in 17 (48.57%) and elevated in 18 (51.43%) patients with SMA, while reduced levels of creatinine were seen in all except 1 of our patients.

All of our 35 patients with SMA and the controls completed the WHOQOL-BREF questionnaire. Twenty-five (71.4%) SMA patients had different comorbidities. These additional medical conditions generally presented with a low degree of severity. We determined conditions affecting the cardiovascular system: hypertension and arrhythmias—tachycardia, bradycardia, or additional cardiac contractions. We determined gastrointestinal problems and nutritional status related to cholelithiasis or a history of cholecystectomy and being overweight/obese (BMI > 25) or underweight (BMI < 18.5). Endocrine disorders in our SMA patients were hypothyroidism and diabetes mellitus. Additionally, we found SMA patients with nephrolithiasis; problems of the skeletal system included scoliosis or severe scoliosis or a history of bone fractures. Some of the patients had undergone orthopedic surgery for scoliosis. The type and frequency of comorbidities in the SMA patients are shown in Table 3.

**Figure 1:**
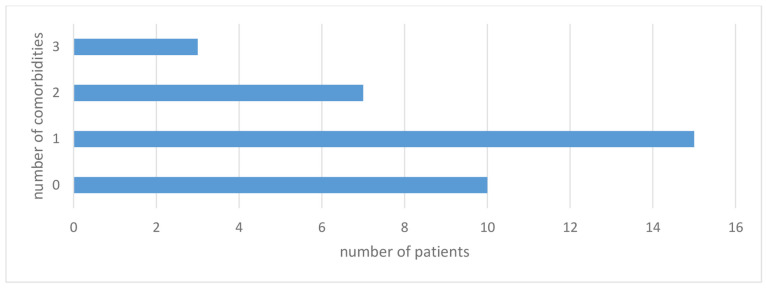
Number of comorbidities in patients with SMA.

The presence of scoliosis or severe scoliosis requiring surgical intervention was primarily treated as a result of alpha motoneuron damage and muscle weakness in the course of the underlying disease [24] and was excluded from the basic calculations regarding comorbidities. Individual patients in the study group were diagnosed with between zero and three conditions other than SMA (Figure 1). The most common disorders associated with SMA were eating disorders in the form of being underweight or overweight (both n = 9, 25.7%).

### 3.2. WHOQOL-BREF Questionnaire Results

#### 3.2.1. WHOQOL-BREF Questionnaire Results for Whole Group of SMA Patients and Control Group

The SMA group had a significantly lower median score for the following questions (see Table 2) compared with the control group: 2 (*p* < 0.001), 3 (*p* = 0.02), 4 (*p* < 0.001), 11 (*p* = 0.008), 17 (*p* = 0.006), 18 (*p* = 0.019), and 21 (*p* = 0.003). In contrast, a significantly higher median score (*p* < 0.001) in the SMA group was obtained for question 24 “How satisfied are you with your access to health services?” The median value for the SMA patients was 4 points and for the control group 3 points, with *p* ≤ 0.001. There were no significant differences between the patient and control groups when comparing the median sum scores for the main domains of the WHOQOL-BREF questionnaire (Table 4). We also did not find any statistical differences in the main domains of the questionnaire depending on gender in the assessed groups.

#### 3.2.2. WHOQOL-BREF Questionnaire Results for Ambulant and Non-Ambulant SMA Patients and Control Group

When we compared non-ambulant patients with the controls, they were better regarding the following questions:

7. “How well are you able to concentrate?” (*p* = 0.031)

9. “How healthy is your physical environment?” (*p* = 0.029)

24. “How satisfied are you with your access to health services?” (*p* < 0.000).

This group achieved lower scores only on the following questions (Table 2): 2 (*p* = 0.001), 4 (*p* < 0.000), and 21 (*p* = 0019). Ambulant patients had similarly higher scores on the WHOQOL-BREF for question 24 (*p* = 0.002). Meanwhile, they had worse results on questions 2 (*p* < 0.000), 4 (*p* < 0.000), 21 (*p* = 0.012), 3 (*p* = 0.034), 10 (*p* = 0.032), 11 (*p* = 0.005), 15 (*p* = 0.005), 17 (*p* = 0.002), and 18 (*p* = 0.01). Non-ambulant patients had significantly higher median scores in comparison with ambulant patients on the following questions: 2 (*p* = 0.037) and 15 (*p* = 0.031).

#### 3.2.3. WHOQOL-BREF Questionnaire Results for Patient Groups with SMA types 2 and 3 and Control Group

A comparison of the questionnaire results in the SMA type 2 and the control groups showed no significant differences, with the exception of question 24. Patients with SMA type 2 as well as type 3 were significantly more satisfied with their access to medical services (for both *p* = 0.03). Significantly more differences were shown when comparing patients with SMA type 3 and healthy people. The scores concerning questions (Table 2) 2 (*p* < 0.000), 4 (*p* < 0.000), 11 (*p* = 0.011), 17 (*p* = 0.003), 18 (*p* = 0.027), and 21 (*p* = 0.004) were statistically lower in the patient group with SMA type 3. Statistically significant differences between patients with SMA type 2 and type 3 were only noted for the following questions: 4 and 14. Scores were higher in the group with SMA type 2 (*p* = 0.037 for both questions).

#### 3.2.4. WHOQOL-BREF Questionnaire Results for SMA Patients with three and four SMN2 Gene Copies

We did not determine any statistical differences among SMA patients with three and four gene copies, except for question number 6 (*p* = 0.015), whose score was higher in patients with three gene copies.

#### 3.2.5. WHOQOL-BREF Questionnaire Results for Patients with SMA in Relation to HFSME Scores

We did not identify any statistical differences in the WHOQOL-BREF questionnaire results between SMA patients with higher and lower HFSME scores.

#### 3.2.6. WHOQOL-BREF Questionnaire Results for Patients with SMA in Relation to Levels of CK

We did not determine any statistical differences in the WHOQOL-BREF questionnaire results between SMA patients with high and normal levels of CK.

#### 3.2.7. WHOQOL-BREF Questionnaire Results for SMA Patients with and without Scoliosis

The comparison of quality of life in groups of SMA patients with and without scoliosis did not reveal any statistically significant differences between the groups.

#### 3.2.8. WHOQOL-BREF Questionnaire Results for SMA Patients with Comorbidities (Other than Scoliosis) and without Comorbidities

The comparison of quality of life in groups of SMA patients with comorbidities other than scoliosis and without comorbidities showed no statistically significant differences.

#### 3.2.9. WHOQOL-BREF Questionnaire Results for SMA Patients with Abnormal Body Mass Index (Overweight or Underweight) and Controls

The comparison of a group of SMA patients who were underweight and the control group did not reveal any significant differences. SMA patients who were overweight obtained statistically significantly lower scores for questions 6 (*p* = 0.028) and 17 (*p* = 0.046). When we compared the results from the questionnaire between groups of overweight and underweight patients, we did not obtain any statistical differences.

#### 3.2.10. Correlations between WHOQOL-BREF Questionnaire Results and Number of Comorbidities in SMA Patients

We did not find any statistically significant correlations between the HSFME or CHOP-INTED scales and the number of comorbidities. Similarly, there were no statistically significant correlations between the questionnaire scores on the main domains and the number of comorbidities (Table 5).

**Figure 2:**
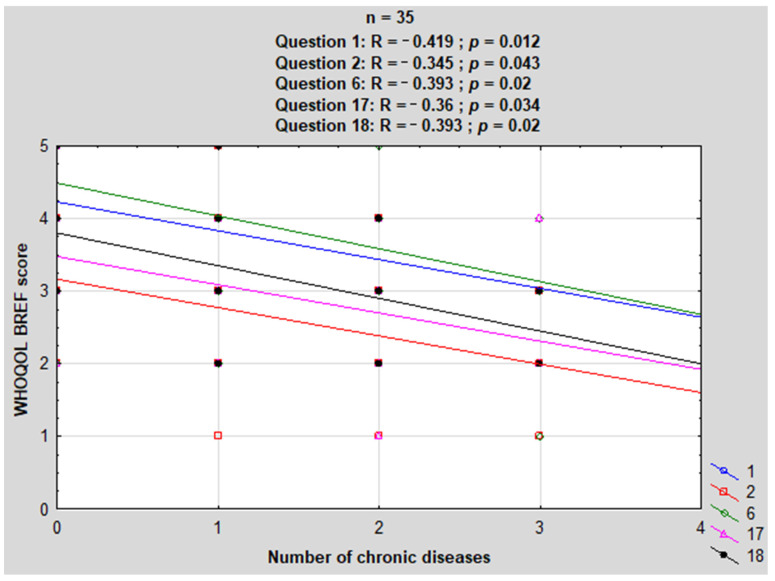
Correlation between the number of comorbidities and scores on questions 1, 2, 6, 17, and 18 on the WHOQOL -BREF scale in SMA patients.

Statistically significant negative correlations were found between the number of comorbidities in SMA patients and the following individual questions on the WHOQOL-BREF questionnaire (Figure 2):

Question 1. “How would you rate your quality of life?”

Question 2. “How satisfied are you with your health?”

Question 6. “To what extent do you feel your life to be meaningful?”

Question 17. “How satisfied are you with your ability to perform your daily living activities?”

Question 18. “How satisfied are you with your capacity for work?”

## 4. Discussion

In our study, we obtained quite unexpected results, partly differing from the literature data.

The most unexpected result of the study is the significantly higher degree of satisfaction with medical care in untreated patients with SMA, regardless of the type of disease, degree of disability, and the presence of comorbidities compared with healthy people, who rated their satisfaction with access to the public health care. In a previous study evaluating SMA patients who transitioned from pediatric to adult health care, widespread unmet health care needs were reported; simultaneously, the SMA patients were proud of their resilience and personal achievements and valued social relationships [25]. In contrast, satisfaction with life, including sex life, bodily appearance, ability to work, and various everyday activities, was significantly lower in the group. Patients indicated that pain and the need for treatment significantly limited their activities. Zamani et al. [5] obtained similar results, i.e., lower QoL in children with SMA than in healthy controls in all domains on the KIDSCREEN-27 questionnaire when conducting a study in a group of children with SMA types 2 and 3. Simultaneously, they did not find any differences between SMA groups.

In the analysis of the questions in the WHOQOL-BREF questionnaire, it was notable that the answers to some of the questions in the patient group with a more severe form of SMA and greater disability (type 2 versus type 3 and walking versus non-walking patients) had significantly higher scores. These results may indicate that patients with a more severe clinical condition and greater disability are more accepting of their limitations and are better equipped to deal with them. In Mix et al. [26], patients with SMA reported high levels of QoL and low levels of depressiveness before starting treatment despite severe physical restrictions. As in our study, early disease onset and low levels of physical functioning were associated with better QoL and lower levels of depressiveness. This may involve a greater role for carers and more active care on their part. Wonhrade et al. [6] used numerous scales and questionnaires to determine the degree of disability and QoL in a group of patients and their caregivers. Their final conclusion was that patient and caregiver well-being levels interact closely.

Other studies [6,27] have shown a relationship different from that in our study: adults and children with low scores on the QoL questionnaires had a more severe phenotype of SMA, e.g., type 1 or 2, only three gene copies, use of wheelchair, scoliosis, and low scores on RULM, HFMSE, etc. Additionally, Thimm et al. [8] showed no improvement in QoL in nusinersen-treated patients who achieved significant improvement in functional scales at a 14-month follow-up.

Based on the results presented in our study and the work by other authors [28], the assessment of the QoL of SMA patients, both following treatment and before the inclusion of therapy, is inconclusive. Discrepancies in the obtained results may be related to the co-occurrence of other factors affecting QoL positively or negatively. These factors may include but are not limited to different comorbidities. Comorbidities can negatively affect the clinical status of SMA patients. In a study in 2427 adults with SMA, Whitney et al. [29] stated that they had noted higher and earlier prevalence of a variety of comorbidities, where the frequency was the highest for the younger age group of 18–40 years, and a relationship with gender was also shown. The most common morbidities present in the female SMA group were hypertension, chronic pulmonary disease, and bone fragility; in the male group, hypertension, cardiac arrhythmias, and diabetes mellitus were recorded.

In studies by Lipnick et al. [30] and Mouchet et al. [31] based on national registries and very large groups of patients (1038 and 1457 subjects, respectively), they demonstrated the presence of non-neuromuscular symptoms, including cardiovascular, gastrointestinal, endocrine, metabolic, reproductive, skeletal, and mental signs, in early SMA. In the above studies, because they were based on databases, the actual clinical status of the patients was not assessed. These studies, therefore, do not show the possible impact of comorbidities on the general condition of SMA patients and consequently on their QoL together with suggested associations with SMN protein deficiency. These problems were very well described in the study by Nery et al. [20]. Impaired renal function was assessed as a frequent comorbidity in patients with SMA type 1. Kidney impairment was probably a consequence of muscular atrophy, immobility, and bone metabolism dysregulation with secondary medullary calcification and tubular dysfunction. SMN deficiency could also influence the expression of different genes encoding proteins important in kidney development and correct renal function. This conclusion complies with those in other studies, e.g., Singh et al.’s [9] and Khawaja et al.’s [32]. A number of authors have described cardiovascular disorders, in the form of congenital heart defects, cardiac arrhythmias, and ECG changes or hypertension [10,17,33,34]. Various metabolic abnormalities in the form of impaired glucose, fatty acid, and amino acid metabolism (and more) with concomitant endocrine dysregulation are also found in SMA patients; this may also result from the denervation process and from the lack of SMN protein [10,14,15,35,36].

We found different comorbidities in our patients, with the most common being over- or underweight. These disorders did not significantly affect the QoL scores. Only overweight patients reported lower self-esteem and lower ability to perform daily activities. In addition, the group of SMA patients with other comorbidities did not differ significantly in their responses compared with the group of SMA patients without other comorbidities. It is worth emphasizing that the above results were obtained despite the fact that up to 10 patients had two or three comorbidities (Figure 1). The results of the study were surprising, as being overweight or obese and having other comorbidities are well known factors in the deterioration in QoL in the context of different chronic diseases [37,38].

The correlation analysis of individual questionnaire questions with the number of comorbidities (from zero to three) showed statistically significant negative correlations in relation to increases in the number of comorbidities. These correlations concerned questions relating primarily to the overall assessment of health and quality of life, reduced self-esteem ability to perform activities of daily living and ability to work (Figure 2). It should be clearly emphasized that the impact of individual comorbidities on quality of life in our SMA patients was not unequivocally negative, as indicated by the questionnaire results discussed earlier. This observation is consistent with reports by other authors collected in a meta-analysis article by Makovski et al. [37].

The strengths of our work were that patients with SMA were personally assessed for disability due to their underlying disease, comorbidities, and their severity. The limitations of the study were the relatively small number of patients compared with database-based cohort studies and the use of only one QoL questionnaire.

## 5. Conclusions

Our study showed that patients with SMA have median sum scores for the main domains of the WHOQOL-BREF questionnaire which are comparable to those of healthy individuals. Patients with more severe forms of SMA scored better than patients with milder forms of SMA on some questions, which may have been due to their better adaptation to the limitations of the disease. There was no significant deterioration in the QoL of SMA patients due to the presence of comorbidities. Only the coexistence of a larger number of comorbidities showed a negative correlation with the patients’ QoL. However, the multiplicity of additional health burdens must have a significant impact on the overall health status of SMA patients, their physical performance, the need for additional treatment, and consequently, the number of necessary contacts with healthcare facilities. All these factors are important for the overall wellbeing of SMA patients and their QoL. This fact is worth remembering, because comorbidities cannot be overlooked in SMA patients, especially in young ones. The late recognition and higher severity of additional diseases might require more intensive therapy with the possibility of developing adverse events. These facts could significantly affect the overall clinical condition of the SMA patient. It seems to be very important that neurologists who deal with SMA patients on a daily basis work closely not only with orthopedists and rehabilitators but also with doctors from different internal medicine specialties, such as diabetologists, pulmonologists, endocrinologists, and nephrologists. As our study shows, the need for numerous contacts with doctors and different treatment centers does not change patients’ positive attitudes towards the need for treatment and their positive opinion of healthcare facilities.

## Figures and Tables

**Table 1:** Demographic and basic clinical data of SMA patients.

	SMA Type 2	SMA Type 3	ALL
Number of patients (%)	5 (14%)	30 (86%)	35
Demographic data			
Age [years]:	25	37.5	35.3
median (Q1–Q3)	(21–34)	(26–49)	(25–44)
Sex			
Women	3	12	15 (43%)
Men	2	18	20 (57%)
Number of SMN2 gene copies			
3 copies	5	16	21 (60%)
4 copies	0	14	14 (40%)
Course of SMA			
Age of SMA onset [years]: median (Q1–Q3)	0.7 (0.7–1)	7.07 (2–7)	3 (2–7)
Number of patients unable to walk	5	14	19 (54.3%)
Number of patients able to walk	0	16	16 (45.7%)
Results on functional scales			
CHOP-INTEND [points]: median (Q1–Q3)	27 (22–27)	46 (30–47) *	39 (27–46) *
HFMSE [points]: median (Q1–Q3)	2 (2–3)	27.5 (9–58)	18 (4–42)

* applies to patients who cannot walk independently (n = 19). SMA—spinal muscular atrophy; SMN—survival of motor neuron; CHOP-INTEND—Children’s Hospital of Philadelphia Infant Test of Neuromuscular Disorders; HFSME—Hammersmith Functional Motor Scale Expanded; Q1–Q3—interquartile ranges.

**Table 2:** The WHOQOL-BREF questionnaire.

WHO-QOL Questions
**Domain 1.** **Physical QOL**
3. To what extent do you feel that (physical) pain prevents you from doing what you need to do?
4. How much do you need any medical treatment to function in your daily life?
10. Do you have enough energy for everyday life?
15. How well are you able to get around?
16. How satisfied are you with your sleep?
17. How satisfied are you with your ability to perform your daily living activities?
18. How satisfied are you with your capacity for work?
**Domain 2 ** **Psychological QOL**
5. How much do you enjoy life?
6. To what extent do you feel your life to be meaningful?
7. How well are you able to concentrate?
11. Are you able to accept your bodily appearance?
19. How satisfied are you with yourself?
26. How often do you have negative feelings such as blue mood, despair, anxiety, depression?
**Domain 3** **Social Relationships QOL**
20. How satisfied are you with your personal relationships?
21. How satisfied are you with your sex life?
22. How satisfied are you with the support you get from your friends?
**Domain 4 ** **Environmental QOL**
8. How safe do you feel in your daily life?
9. How healthy is your physical environment?
12. Have you enough money to meet your needs?
13. How available to you is the information that you need in your day-to-day life?
14. To what extent do you have the opportunity for leisure activities?
23. How satisfied are you with the conditions of your living place?
24. How satisfied are you with your access to health services?
25. How satisfied are you with your transport?
**General QOL**
1. How would you rate your quality of life?
2. How satisfied are you with your health?

**Table 3:** The type and frequency of comorbidities in the SMA patients.

Comorbidities
**Cardiovascular system**
Hypertension
Arrhythmia (tachycardia/bradycardia)
**Gastrointestinal system/nutritional status**
Overweight/obesity (BMI > 25)
Underweight (BMI < 18.5)
Cholelithiasis/cholecystectomy
**Hormonal system**
Hypothyroidism
Diabetes mellitus type 2/insulin resistance
**Urinary system**
Nephrolithiasis
**Skeletal system**
Bone fractures
Scoliosis/scoliosis surgery

**Table 4:** The results as median sum scores on the WHOQOL-BREF questionnaire for the main domains in the SMA and control groups.

WHOQOL-BREF	SMA				Controls				*p*
	n = 35				n = 36				
Domains	Range	Q1	M	Q3	Range	Q1	M	Q3	
**Physical**	14–29	20	23	25	16–27	19.5	21.5	24	0.300
**Mental**	14–26	19	22	24	15–27	21	22.5	24	0.440
**Social**	7–15	10	12	13	8–15	10.5	12	13	0.461
**Environmental**	20–40	27	30	32	23–36	26	29	32	0.328

SMA—spinal muscular atrophy; WHOQOL-BREF—World Health Organization Quality of Life Brief Version; Q1—first quartile; Q3—third quartile; M—median; n—number of patients.

**Table 5:** The correlation between the number of comorbidities and scores on the main domains on the WHOQOL-BREF questionnaire in 35 SMA patients.

WHOQOL-BREF Domains	R	*p*
Physical domain and number of comorbidities	−0.234564	0.174999
Mental domain and number of comorbidities	−0.255117	0.139119
Social domain and number of comorbidities	−0.138285	0.428239
Environmental domain and number of comorbidities	−0.299816	0.080148

SMA—spinal muscular atrophy; WHOQOL-BREF—World Health Organization Quality of Life Brief Version; R—Spearman rank correlation coefficient.

## Data Availability

All data are available to other investigators for the purpose of replication and re-use upon request. Contact person: Magdalena Koszewicz (e-mail: magdalena.koszewicz@umw.edu.pl).
